# Brain magnetic resonance imaging in imported malaria

**DOI:** 10.1186/s12936-019-2713-2

**Published:** 2019-03-12

**Authors:** Andreas M. Frölich, Pinkus Tober-Lau, Michael Schönfeld, Thomas T. Brehm, Florian Kurth, Christof D. Vinnemeier, Marylyn M. Addo, Jens Fiehler, Thierry Rolling

**Affiliations:** 1 Department of Diagnostic and Interventional Neuroradiology, University Medical Centre Hamburg-Eppendorf, Hamburg, Germany; 2 Department of Infectious Diseases and Pulmonary Medicine, Charité-Universitätsmedizin Berlin, Corporate Member of Freie Universität Berlin, Humboldt-Universität zu Berlin, and Berlin Institute of Health, Charitéplatz 1, 10117 Berlin, Germany; 3 Divisions of Infectious Diseases and Tropical Medicine, I. Department of Internal Medicine, University Medical Centre Hamburg-Eppendorf, Hamburg, Germany; 4 Clinical Research Department, Bernhard-Nocht-Institute for Tropical Medicine, Hamburg, Germany; 5 Department for Clinical Immunology of Infectious Diseases, Bernhard-Nocht-Institute for Tropical Medicine, Hamburg, Germany

**Keywords:** Malaria, Imported malaria, Cerebral malaria, MRI, *P. falciparum*, Complicated malaria, Uncomplicated malaria, Splenium, Splenial lesion

## Abstract

**Background:**

Previous studies have documented a spectrum of brain magnetic resonance imaging (MRI) abnormalities in patients with cerebral malaria, but little is known about the prevalence of such abnormalities in patients with non-cerebral malaria. The aim of this study was to assess the frequency of brain MRI findings in returning travellers with non-cerebral malaria.

**Methods:**

A total of 17 inpatients with microscopically confirmed *Plasmodium falciparum* non-cerebral malaria underwent structural brain MRI at 3.0 Tesla, including susceptibility-weighted imaging (SWI). Presence of imaging findings was recorded and correlated with clinical findings and parasitaemia.

**Results:**

Structural brain abnormalities included a hyperintense lesion of the splenium on T2-weighted imaging (n = 3) accompanied by visible diffusion restriction (n = 2). Isolated brain microhaemorrhage was detected in 3 patients. T2-hyperintense signal abnormalities of the white matter ranged from absent to diffuse (n = 10 had 0–5 lesions, n = 5 had 5–20 lesions and 2 patients had more than 50 lesions). Imaging findings were not associated with parasitaemia or HRP2 levels.

**Conclusion:**

Brain MRI reveals a considerable frequency of T2-hyperintense splenial lesions in returning travellers with non-cerebral malaria, which appears to be independent of parasitaemia.

## Background

Malaria is a systemic parasitic disease involving multiple organ systems in severe cases. Patients present with a spectrum of clinical syndromes ranging from the mild to the life-threatening. Cerebral malaria, defined as impaired consciousness (Glasgow Coma Scale score < 11) in the absence of another cause than malaria, accounts for a large part of morbidity and mortality in the acute phase of severe malaria. Pathophysiologically, it is thought to be at least partially related to the sequestration of infected erythrocytes in the microvasculature of the brain with ensuing obstruction and hypoperfusion [1, 2]. However, it is unlikely that parasite sequestration and involvement of the brain is present only in patients with strictly defined cerebral malaria. It seems that sequestration in the cerebral and other microvasculature also occurs in other forms of severe and even uncomplicated malaria along a continuum with only the highest sequestered biomass being seen in cerebral malaria [3, 4]. So far, most investigations describing brain MRI have focused on patients with cerebral malaria. In adults, relatively few case reports have described the occurrence of a splenial lesion in patients with cerebral malaria [5–8]. Only one manuscript has assessed cerebral involvement in uncomplicated malaria and also described transient lesions in the splenium as distinct findings [9]. In addition, few reports suggest that susceptibility-weighted imaging (SWI), an imaging technique highly sensitive to microhaemorrhages, may reveal brain microhaemorrhages in patients with cerebral malaria [10, 11]. Imported malaria differs from malaria cases in endemic regions regarding several aspects. Affected patients are mainly adults with no or waning prior anti-malarial immunity [12]. Asymptomatic chronic parasitaemia is rare, as are coinfections such as bacteraemia and nutrient deficiencies [13]. Due to the absence of these factors, returning travellers with malaria form a more homogeneous population in which to investigate cerebral involvement by magnetic resonance imagin (MRI) without a confounding effect.

The aim of this study was to assess the frequency of cerebral microhaemorrhages and other brain MRI findings in returning travellers with malaria and to assess whether these findings are associated with parasitaemia and of clinical disease along the spectrum of mild to complicated malaria.

## Methods

### Patients

Recruitment took place between December 2014 and October 2016 at the University Medical Centre Hamburg–Eppendorf. Inpatients with microscopically-confirmed *Plasmodium falciparum* malaria were asked to participate in the study. Patients had to be fluent in German or English and be residents of Germany to be included in the study. This would provide the basis for informed consent and give the possibility to follow-up patients if any incidental finding would have been detected on cerebral MRI. Unconscious patients could not be included in the study. Obese patients as well as those with claustrophobia were excluded due to the constraints of the available MRI. Patients were treated according to the discretion of the responsible physician, and this decision was independent of any study participation.

### Parasitaemia and HRP2 measurements

Peripheral parasitaemia was determined on drawn venous blood by standard World Health Organization (WHO) microscopy techniques at the Bernhard-Nocht-Institute for Tropical Medicine. PfHRP2 was measured by double site sandwich ELISA. ELISA plates (F96 CERT.Maxisorp Nunc-Immuno plate, Thermo Fisher Scientific Inc., Waltham, MA, USA) were incubated at 4 °C over night with primary IgM antibody (MBS563506, MyBioSource, Inc., San Diego, CA, USA) diluted to 1 µg/ml in 2% BSA and 98% PBS and then washed with 0.1% PBS/Tween20 (PBST). Samples were pre-diluted in PBST (depending on parasitaemia 1:2, 1:10 or 1:100) and then 3 more times in dilution series (1:2) along with a PfHRP2 standard dilution series starting at 10 ng/ml (kindly provided by DJ Sullivan, Johns Hopkins Bloomberg School of Public Health, Baltimore, MD), transferred in doubles to the pre-coated plate (100 µl/well) and incubated for 1 h at room temperature. After washing 100 µl of secondary IgG antibody (MBS563505, MyBioSource, Inc., San Diego, CA, USA) diluted to 0.2 µg/ml were transferred to each well, incubated for 1 h at room temperature and washed. Finally, 100 µl of TMB chromogen (TMB ELISA Substrate Solution, eBiosciences, Inc., San Diego, CA, USA) were transferred to each well, incubated for 5 min and the reaction stopped with 50 µl 1 M sulfuric acid. Extinction measurement was performed at 450 nm with FilterMax F5 (Molecular Devices, LLC, San Jose, CA, USA) and analysed with SoftMax Pro 6.3 (Molecular Devices, LLC, San Jose, CA, USA) and Microsoft Excel. Samples lying outside the linear standard range were re-analysed at higher dilution.

### Brain MRI acquisition

The goal was to perform the MRI within 24 h after anti-malarial treatment was initiated. If this was not feasible, patients were still included in the study if the MRI could be performed within 48 h after treatment was initiated.

Brain MRI was acquired using a 3 Tesla Magnetom Skyra (Siemens, Erlangen, Germany). The protocol (Table 1) included 3D fluid-attenuated inversion recovery (FLAIR), 3D T2 turbo spin echo (T2w), 3D gradient-recalled T1 weighted imaging (T1w), axial susceptibility-weighted imaging (SWI) and axial intravoxel incoherent motion diffusion weighted imaging (DWI) with calculation of maps of the apparent diffusion coefficient (ADC). No contrast medium was administered.

**Table 1:** MR sequence characteristics

Sequence	Repetition time in ms	Echo time in ms	Inversion time in ms	Flip angle	Slice thickness in mm
3D flair	4700	392	1800	120	0.9
SWI	37	25	–	15	1.5
DWI	4700	85	–	90	3.0
MPRAGE	1900	2.5	900	9	0.9

### Image analysis

Brain MRI was independently assessed for structural lesions by two radiologists (AF, MS) blinded to clinical information. Disagreements were settled by consensus. One radiologist (AF) counted the total number of hyperintense white matter lesions on 3D FLAIR imaging as well as the total number of punctate intracerebral hypointense lesions corresponding to microhaemorrhages on SWI.

To measure the ADC in different brain regions, circular ROIs were manually placed by a radiologist (AF) on ADC maps in the following locations: Bilateral posterior limb of the internal capsule and corona radiata as well as the central genu and splenium of the corpus callosum.

### Statistical analysis

Variables are reported using standard descriptive statistics. ADC values were compared to normative values using an independent *t* test. Pearson correlation was used to assess for associations between ADC values and laboratory findings. The association between the occurrence of a splenial lesion or of microhaemorrhages with parasitaemia and HRP2 levels was assessed by Wilcoxon rank sum test.

## Results

A total of 17 patients underwent MRI and were included in the analysis (Table 2). Half (n = 8) were immigrants from malaria-endemic countries who were visiting friends and relatives (VFR), while the other half were travellers not originating from malaria-endemic countries. Only three patients were women. Patients’ age ranged between 20 and 64 years. One patient additionally had type II diabetes, one had known hypertension, and one had been treated for non-Hodgkin lymphoma 5 years prior to the malaria diagnosis. None of the others had any relevant comorbidities.

**Table 2:** Baseline characteristics of included participants

N	17
Male sex, n (%)	14 (82%)
Age, years	49 (20–64)
Visiting friends and relatives, n(%)	8 (47%)
Percentage of infected erythrocytes	1% (0.1–40%)
Patients with complicated malaria, n (%)	5 (31%)
Hyperparasitaemia (> 10%)	4
Shock, needing vasopressor support	1
Spontaneous bleeding	1
Haemoglobinuria	1
Acute kidney injury	1
GCS at presentation (median, range)	15 (12–15)
Neurological symptoms at presentation, n(%)	
Headaches	7 (41%)
Aphasia	2 (12%)
Disorientation	2 (12%)
Somnolence	1 (6%)
Haemoglobin at presentation, g/dl	13.1 (10.3–15.7)
White blood count at presentation, /µl	4.8 (3.7–10.3)
Thrombocyte count at presentation, /µl	61 (11–209)
Treatment regimens, n (%)	
Artesunate, followed by Atovaquone/proguanil	5 (29%)
Dihydroartemisinin/piperaquine	6 (35%)
Atovaquone/proguanil	5 (29%)
Mefloquine	1 (6%)

Values represent median and range, unless otherwise specified

Severity of malaria ranged from very mild (with few clinical symptoms and less than 0.1% of infected erythrocytes) to severe (with neurological involvement and very high parasitaemia of 40% of infected erythrocytes). Formally, five patients were classified as complicated malaria according to WHO criteria [14]. Three of these patients with complicated malaria and one patient with formally uncomplicated malaria had reduced vigilance and/or confusion (Glasgow Coma Scale scores between 12 and 14), but did not meet the criteria of cerebral malaria, as defined by the WHO [14, 15]. No focal neurological deficits were noted on neurological examination of any of the included patients.

### Brain MRI findings

**Fig. 1:**
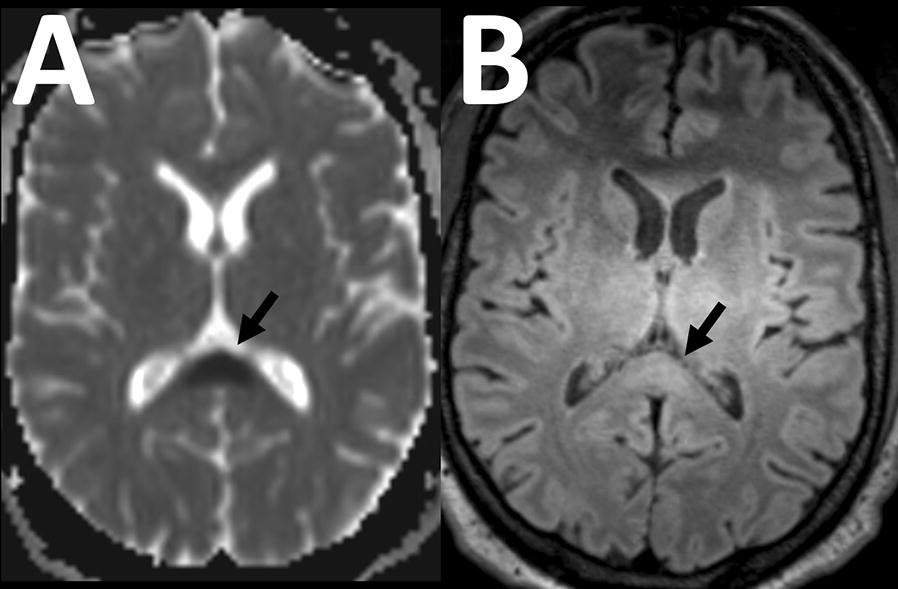
Splenial lesion. In a 39-year old male patent, axial apparent diffusion coefficient map from diffusion weighted imaging **A** shows a focal lesion in the splenium of the corpus callosum with strong diffusion restriction (arrow). There is slight accompanying signal hyperintensity on the corresponding Flair image (**B**). Slight hyperintensity of the bilateral thalami was uniformly seen on our scanner with this sequence and considered artefactual

Structural brain abnormalities detected included a hyperintense lesion of the splenium on T2-weighted imaging (n = 3), accompanied by visible diffusion restriction in two cases (Fig. 1). An isolated single brain microhaemorrhage was detected in 3 patients. Small focal T2-hyperintense signal abnormalities of the deep and periventricular white matter were frequent findings, ranging from absent to diffuse (n = 10 had 0–5 lesions, n = 5 had 5–20 lesions and 2 patients had more than 50 lesions).

Mean ADC values for the different locations are displayed in Table 3. Neither parasitaemia nor HRP2 levels correlated with the ADC in any of the locations (p > 0.05 for all). In patients with a splenial lesion (n = 3), the ADC measured in the splenium was significantly lower than in those without a splenial lesion (0.351 ± 0.147 * 10^−3^ mm^2^/s vs. 0.682 ± 0.074 * 10^−3^ mm^2^/s; p < 0.001), while the ADC in all other measured locations was not significantly different between these groups (p > 0.05 for all, Table 3). The occurrence of a splenial lesion was not associated with parasitaemia (median parasitaemia of 1.5% infected erythrocytes in patients without a splenial lesion vs. 1.0% in those with a splenial lesion, p = 0.231) or HRP2 levels (median HRP2 levels of 27 ng/ml in patients without a splenial lesion vs. 1 ng/ml in those with a splenial lesion, p = 0.142).

**Table 3:** Apparent diffusion coefficient values in patients with and without T2-hyperintense splenial lesions

Location	Mean apparent diffusion coefficient (10^−3^ mm^2^/s)		
	Splenial lesion absent	Splenial lesion present	
Corpus callosum: Splenium	682 ± 74	351 ± 147	p < 0.001
Corpus callosum: Genu	691 ± 56	682 ± 30	p = 0.84
Corona radiata: left	646 ± 39	632 ± 26	p = 0.64
Corona radiata: right	627 ± 45	627 ± 9	p = 0.98
Internal capsule: left	660 ± 67	684 ± 14	p = 0.64
Internal capsule: right	604 ± 51	641 ± 38	p = 0.35

Values represent mean and standard deviation

None of the patients with a splenial lesion had hyperparasitaemia, acute kidney injury, severe anaemia, decreased vigilance or confusion. Timing of MRI was similar for patients with a splenial lesion and those without a splenial lesion (median time of 18 h for those without a lesion and 23 h for those with a lesion, p = 0.1306). Furthermore parasitaemia did not correlate with the number of white matter lesions (r = − 0.22; p = 0.41). There was no association between the occurrence of a microhaemorrhage and parasitaemia (median parasitaemia of 1% in both groups, p = 0.719). or HRP2 levels (median HRP2 levels of 26 ng/ml in patients without a microhaemorrhage vs. 1 ng/nl in patients with a single microhaemorrhages, p = 0.051).

## Discussion

The present study describes the frequency of brain MRI abnormalities in a cohort of returning travellers with non-cerebral malaria. Over 80% of study participants were male, which is in line with national notification reports for malaria, which show a three-time higher malaria incidence in male returning travellers to Germany [16]. Clinically silent splenial lesions were seen in moderate frequency and were in part accompanied by diffusion restriction. Similar splenial lesions were detected at an even higher frequency in a cohort of children with a clinical definition of cerebral malaria in an endemic area [17] as well as in smaller series of uncomplicated endemic malaria [9]. In the present cohort, splenial lesions also occur in returning travellers with non-cerebral malaria with a potentially lower frequency in this specific population. Possibly, this lower frequency may be related to the lower rate of comorbidities, such as bacteraemia, in the target population compared to endemic malaria. Additionally, MRI was not performed concurrently to treatment start. While there was no association between MRI timing and occurrence of splenial lesions, some less pronounced lesions may have been missed due to late MRI.

In the absence of a correlation between parasitaemia or HRP2 levels and the presence of splenial lesions, the occurrence of such lesions may not be directly linked to parasitaemia of infected erythrocytes but may rather be an epi-phenomenon. On the other hand, the higher prevalence of splenial involvement observed in more severely affected children with cerebral malaria [17] would suggest a link to disease severity. Possible explanations include the modest sample size in the current study as well as the restriction to patients with non-cerebral malaria due to ethical.

Given the sample of patients with splenial lesions and ensuing low power, more data are needed to assess whether this imaging finding may carry prognostic implications. In the setting of acute malarial infection, it might be theoretically conceivable that the presence of early brain imaging findings could help better predict patients at risk of developing complicated or cerebral malaria. However, due to the small sample and lack of follow-up, this was beyond the scope of the present study.

Few previous studies have reported the use of SWI in the context of malaria, describing brain microhaemorrhages in one adult and in sixteen children with cerebral malaria [10, 11]. Using this highly sensitive technique, the frequency of such microhaemorrhages in returning travellers with non-cerebral malaria is low and may overlap with the range expected in the normal population. For comparison, one of the largest population-based studies (the Rotterdam scan study) showed cerebral microbleeds in 17.8% of the general population aged 60–69 years and 38.3% in those over 80 years compared to 17.6% in the study population (median age 49 years). Comparison of these results is complicated by differences in MRI technique, age and comorbidities. While study patients were younger (which should decrease the number of microbleeds observed), SWI was used, which increases sensitivity for cerebral microbleeds compared to the T2*-weighted gradient-recalled echo sequences [18] that have been utilized in population-based studies including the Rotterdam study [19]. The few microhaemorrhages that were detected did not show a correlation with parasitaemia or clinical symptoms. Furthermore, no patient demonstrated more than a single microbleed, and the diagnostic utility of a single isolated microhaemorrhage has previously been questioned [20]. These findings argue against the hypothesis that clinically silent cerebral microbleeds occur at an increased frequency in returning travellers with malaria and in low parasitaemia. However, these results cannot be readily translated to sequestered infected erythrocytes. As these would be expected to remain intravascularly (in contrast to cerebral microbleeds, which require extravasation of erythrocytes), any sequestered infected erythrocytes were likely too small or too mobile to be detected by the imaging protocol. On the other hand, it cannot be excluded that sequestered erythrocytes may in fact be detected by SWI but were simply not present in the study patient’s cerebral circulation, although this seems unlikely. Further optimization of the SWI imaging protocol or imaging at even higher field strengths may increase the sensitivity for detection of sequestered parasitized erythrocytes.

T2-hyperintense foci of white matter signal abnormality were detected in most patients and ranged from absent to extensive, with most patients displaying no or very limited white matter changes. Large population-based studies have found a considerable frequency of such lesions and have correlated the number and extent of such lesions with age and vascular risk factors, commonly suggesting a microvascular origin of these signal changes [21]. The number of such lesions did not correlate with parasitaemia. Although the study design does not allow to rule out a connection of these white matter changes with malarial infection, it is most likely that these changes are of microvascular aetiology and consider them incidental lesions.

## Limitations

Since the primary study hypothesis concerned the detection of clinically silent cerebral microhaemorrhages, a routine follow-up MRI examination was not included in the study design. Follow-up MRI was recommended to all participants with a splenial lesion to check for resolution, but results were not available due to loss of these patients to follow-up. Previous studies have shown reversibility of splenial lesions observed in malaria patients [5, 6, 9].

## Conclusions

Brain MRI reveals a moderate frequency of T2-hyperintense splenial lesions in returning travellers with non-cerebral malaria, which appears to be independent of parasitaemia. More data are needed to assess whether this imaging finding may have clinical or prognostic implications.
